# Supplementary figures and images for: Profiling serum immunodominance following SARS-CoV-2 primary and breakthrough infection reveals distinct variant-specific epitope usage and immune imprinting
Source: PLoS Pathog. 2024 Nov 18;20(11):e1012724. doi: 10.1371/journal.ppat.1012724 (PMC11611254; doi:10.1371/journal.ppat.1012724)

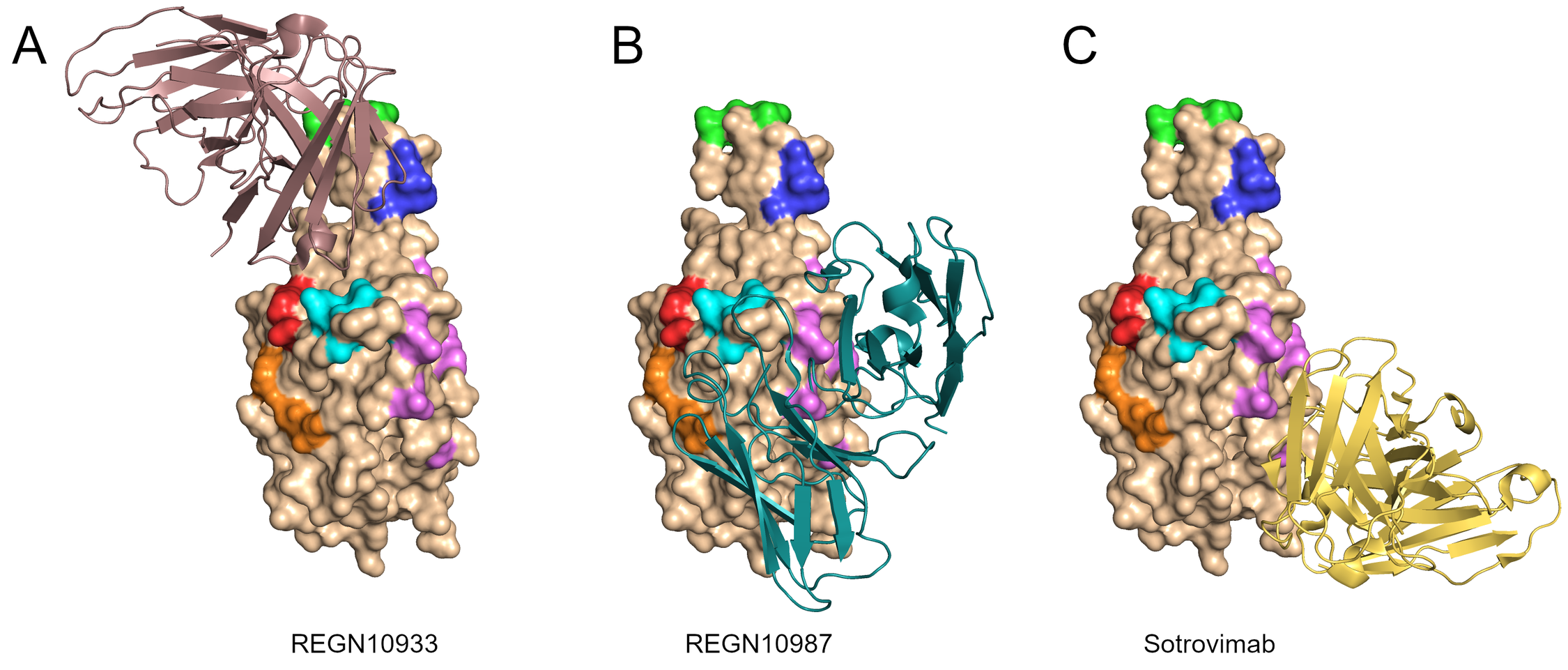

Supplement: S1 Fig — Structures of therapeutic monoclonal antibodies in complex with RBD; (A) REGN10933 (PDB: 6XDG), (B) REGN10987 (PDB: 6XDG) and (C) Sotrovimab (PDB: 6WPS). Fabs (cartoon representation) are binding to SARS-CoV-2 RBD (surface representation) with revertant residues coloured as in Fig 1C and S2 Table. (TIF) [file ppat.1012724.s004.tif]

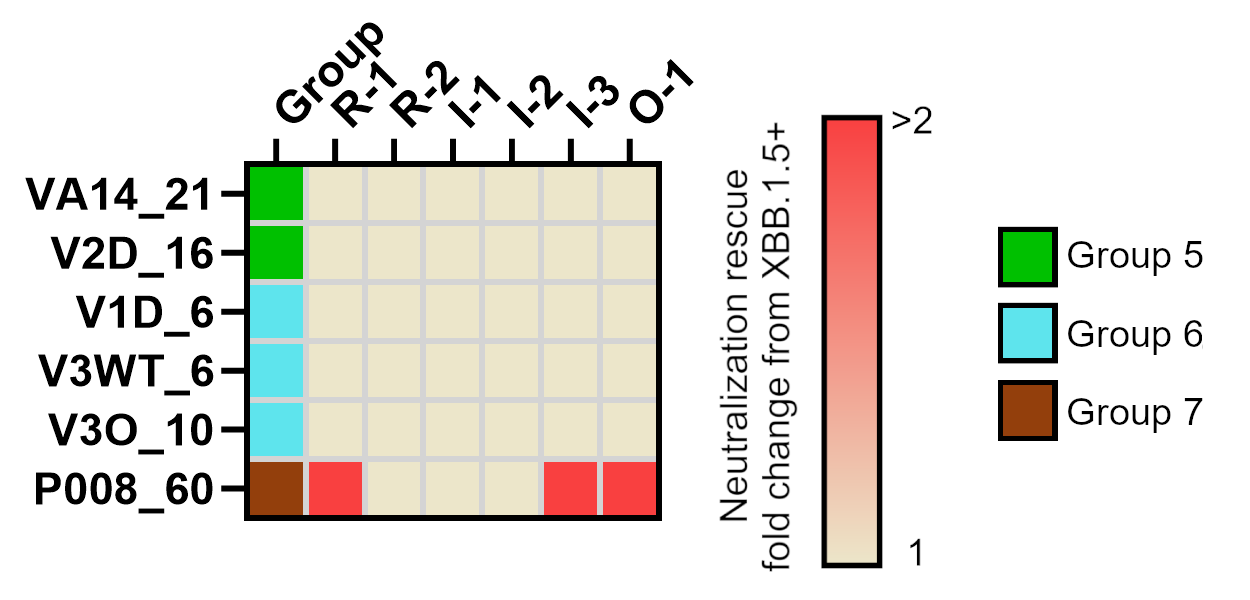

Supplement: S2 Fig — (TIF) [file ppat.1012724.s005.tif]

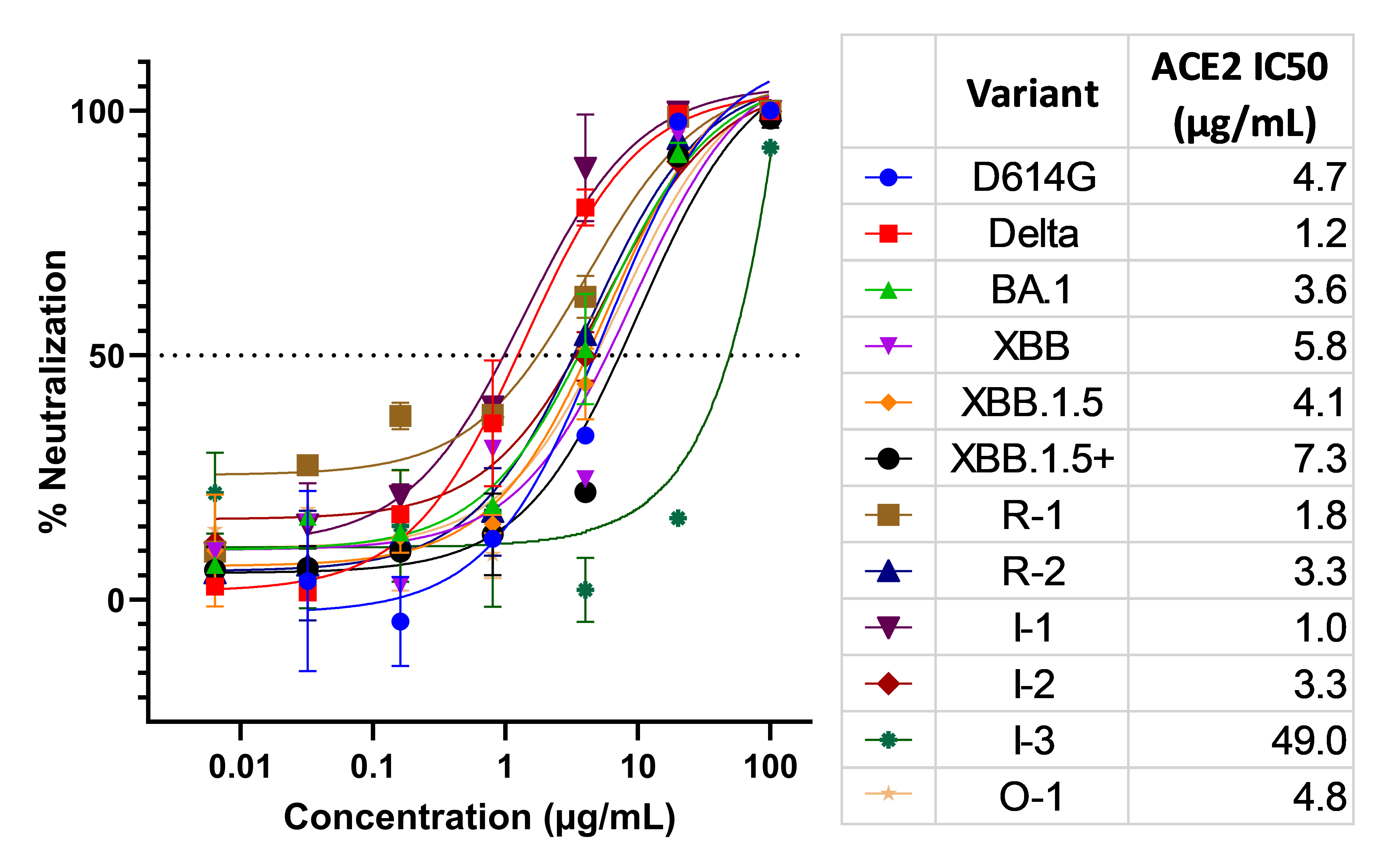

Supplement: S3 Fig — Spikes with higher binding affinity are neutralized more efficiently by soluble ACE2 Ref 28. A table of ACE2 IC50 values for each spike is included in the legend. (TIF) [file ppat.1012724.s006.tif]

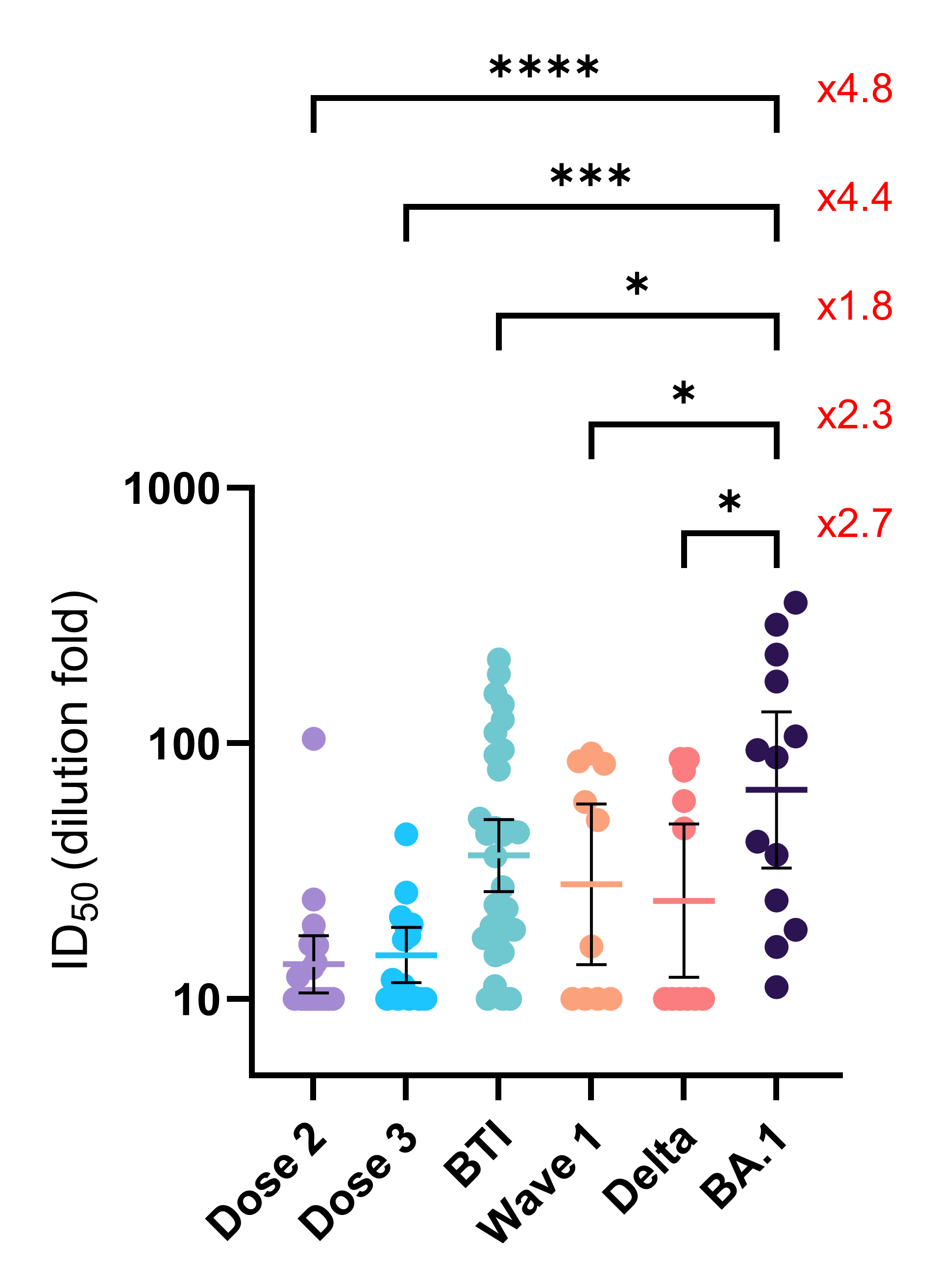

Supplement: S4 Fig — Geometric mean ID50 are indicated by horizontal bars and the fold change compared to BA.1 primary infection sera is shown in red text. Error bars indicate geometric mean ± s.d. (TIF) [file ppat.1012724.s007.tif]

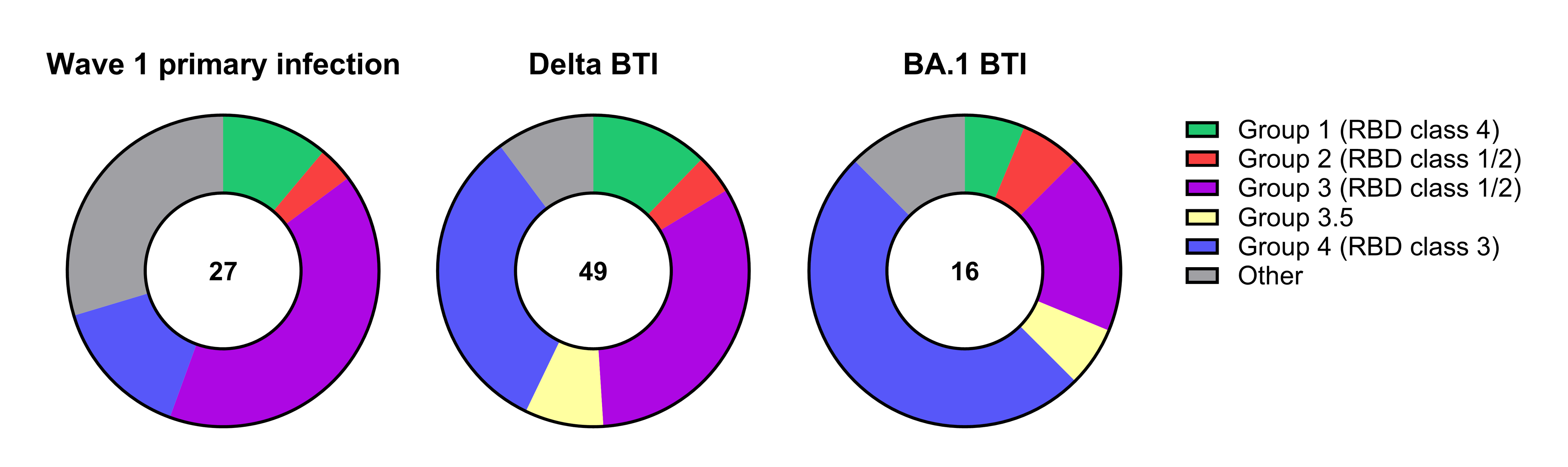

Supplement: S5 Fig — Total number of mAbs analysed is shown within the pie chart. RBD groups have been defined previously in Graham et al. [Ref 2] and matching RBD epitope classes have defined by Barnes et al. [Ref 5]. (TIF) [file ppat.1012724.s008.tif]
